# Supplementary material for: Autoinhibited kinesin-1 adopts a hierarchical folding pattern
Source: eLife. 2023 Nov 1;12:RP86776. doi: 10.7554/eLife.86776 (PMC10619981; doi:10.7554/eLife.86776)
Supplement: Figure 5—source data 2. [file elife-86776-fig5-data2.zip › KIF5B_IAK_AAA/KIF5B_IAK_AAA_seq.rtf]

>Kif5b_IAKMADLAECNIKVMCRFRPLNESEVNRGDKYIAKFQGEDTVVIASKPYAFDRVFQSSTSQEQVYNDCAKKIVKDVLEGYNGTIFAYGQTSSGKTHTMEGKLHDPEGMGIIPRIVQDIFNYIYSMDENLEFHIKVSYFEIYLDKIRDLLDVSKTNLSVHEDKNRVPYVKGCTERFVCSPDEVMDTIDEGKSNRHVAVTNMNEHSSRSHSIFLINVKQENTQTEQKLSGKLYLVDLAGSEKVSKTGAEGAVLDEAKNINKSLSALGNVISALAEGSTYVPYRDSKMTRILQDSLGGNCRTTIVICCSPSSYNESETKSTLLFGQRAKTIKNTVCVNVELTAEQWKKKYEKEKEKNKILRNTIQWLENELNRWRNGETVPIDEQFDKEKANLEAFTVDKDITLTNDKPATAIGVIGNFTDAERRKCEEEIAKLYKQLDDKDEEINQQSQLVEKLKTQMLDQEELLASTRRDQDNMQAELNRLQAENDASKEEVKEVLQALEELAVNYDQKSQEVEDKTKEYELLSDELNQKSATLASIDAELQKLKEMTNHQKKRAAEMMASLLKDLAEIGIAVGNNDVKQPEGTGMIDEEFTVARLYISKMKSEVKTMVKRCKQLESTQTESNKKMEENEKELAACQLRISQHEAKIKSLTEYLQNVEQKKRQLEESVDALSEELVQLRAQEKVHEMEKEHLNKVQTANEVKQAVEQQIQSHRETHQKQISSLRDEVEAKAKLITDLQDQNQKMMLEQERLRVEHEKLKATDQEKSRKLHELTVMQDRREQARQDLKGLEETVAKELQTLHNLRKLFVQDLATRVKKSAEIDSDDTGGSAAQKQKISFLENNLEQLTKVHKQLVRDNADLRCELPKLEKRLRATAERVKALESALKEAKENASRDRKRYQQEVDRIKEAVRSKNMARRGHSAQAAAPIRPGQHPAASPTHPSAIRGGGAFVQNSQPVAVRGGGGKQVGSGSGLEVLFQGPLEVLFQGPGSGSGMVSKGEAVIKEFMRFKVHMEGSMNGHEFEIEGEGEGRPYEGTQTAKLKVTKGGPLPFSWDILSPQFMYGSRAFTKHPADIPDYYKQSFPEGFKWERVMNFEDGGAVTVTQDTSLEDGTLIYKVKLRGTNFPPDGPVMQKKTMGWEASTERLYPEDGVLKGDIKMALRLKDGGRYLADFKTTYKAKKPVQMPGAYNVDRKLDITSHNEDYTVVEQYERSEGRHSTGGMDELYKLEWSHPQFEK
