# Supplementary material for: Autoinhibited kinesin-1 adopts a hierarchical folding pattern
Source: eLife. 2023 Nov 1;12:RP86776. doi: 10.7554/eLife.86776 (PMC10619981; doi:10.7554/eLife.86776)
Supplement: Figure 6—source data 1. [file elife-86776-fig6-data1.zip › TRAK1_KIF5B/TRAK1_KIF5B_seq.rtf]

Kif5bMADLAECNIKVMCRFRPLNESEVNRGDKYIAKFQGEDTVVIASKPYAFDRVFQSSTSQEQVYNDCAKKIVKDVLEGYNGTIFAYGQTSSGKTHTMEGKLHDPEGMGIIPRIVQDIFNYIYSMDENLEFHIKVSYFEIYLDKIRDLLDVSKTNLSVHEDKNRVPYVKGCTERFVCSPDEVMDTIDEGKSNRHVAVTNMNEHSSRSHSIFLINVKQENTQTEQKLSGKLYLVDLAGSEKVSKTGAEGAVLDEAKNINKSLSALGNVISALAEGSTYVPYRDSKMTRILQDSLGGNCRTTIVICCSPSSYNESETKSTLLFGQRAKTIKNTVCVNVELTAEQWKKKYEKEKEKNKILRNTIQWLENELNRWRNGETVPIDEQFDKEKANLEAFTVDKDITLTNDKPATAIGVIGNFTDAERRKCEEEIAKLYKQLDDKDEEINQQSQLVEKLKTQMLDQEELLASTRRDQDNMQAELNRLQAENDASKEEVKEVLQALEELAVNYDQKSQEVEDKTKEYELLSDELNQKSATLASIDAELQKLKEMTNHQKKRAAEMMASLLKDLAEIGIAVGNNDVKQPEGTGMIDEEFTVARLYISKMKSEVKTMVKRCKQLESTQTESNKKMEENEKELAACQLRISQHEAKIKSLTEYLQNVEQKKRQLEESVDALSEELVQLRAQEKVHEMEKEHLNKVQTANEVKQAVEQQIQSHRETHQKQISSLRDEVEAKAKLITDLQDQNQKMMLEQERLRVEHEKLKATDQEKSRKLHELTVMQDRREQARQDLKGLEETVAKELQTLHNLRKLFVQDLATRVKKSAEIDSDDTGGSAAQKQKISFLENNLEQLTKVHKQLVRDNADLRCELPKLEKRLRATAERVKALESALKEAKENASRDRKRYQQEVDRIKEAVRSKNMARRGHSAQIAKPIRPGQHPAASPTHPSAIRGGGAFVQNSQPVAVRGGGGKQV Trak1(1-395)DKDCEMKRTTLDSPLGKLELSGCEQGLHRIIFLGKGTSAADAVEVPAPAAVLGGPEPLMQATAWLNAYFHQPEAIEEFPVPALHHPVFQQESFTRQVLWKLLKVVKFGEVISYSHLAALAGNPAATAAVKTALSGNPVPILIPCHRVVQGDLDVGGYEGGLAVKEWLLAHEGHRLGKPGLGGSGSGSMALVFQFGQPVRAQPLPGLCHGKLIRTNACDVCNSTDLPEVEIISLLEEQLPHYKLRADTIYGYDHDDWLHTPLISPDANIDLTTEQIEETLKYFLLCAERVGQMTKTYNDIDAVTRLLEEKERDLELAARIGQSLLKKNKTLTERNELLEEQVEHIREEVSQLRHELSMKDELLQFYTSAAEESEPESVCSTPLKRNESSSSVQNYFHLDSLQKKLKDLEEENVVLRSEASQLKTETITYEEKEQQLVNDCVKELRDANVQIASISEELAKKTEDAARQQEEITHLLSQIVDLQKKAKACAVENEELVQHLGAAKDAQRQLTAELRELEDKYAECMEMLHEAQEELKNLRNKTMPNTTSRRYHSLGLFPMDSLAAEIEGTMRKELQLEEAESPD
